# Factors associated with pastoral community knowledge and occurrence of mycobacterial infections in Human-Animal Interface areas of Nakasongola and Mubende districts, Uganda

**DOI:** 10.1186/1471-2458-10-471

**Published:** 2010-08-10

**Authors:** Clovice Kankya, Adrian Muwonge, Susan Olet, Musso Munyeme, Demelash Biffa, John Opuda-Asibo, Eystein Skjerve, James Oloya

**Affiliations:** 1 Department of Veterinary Public Health, Faculty of Veterinary Medicine Makerere University, P.O. Box 7062, Kampala, Uganda; 2 Centre for Epidemiology and Biostatistics, Norwegian School of Veterinary Science, P.O. Box 8146 Dep., 0033 Oslo, Norway; 3 Department of Disease Control, School of Veterinary Medicine, University of Zambia, P.O BOX 32379, Lusaka Zambia

## Abstract

**Background:**

Nontuberculous mycobacteria (NTM) are emerging opportunistic pathogens whose role in human and animal disease is increasingly being recognized. Major concerns are their role as opportunistic pathogens in HIV/AIDS infections. The role of open natural water sources as source and livestock/wildlife as reservoirs of infections to man are well documented. This presents a health challenge to the pastoral systems in Africa that rely mostly on open natural water sources to meet livestock and human needs. Recent study in the pastoral areas of Uganda showed infections with same genotypes of NTM in pastoralists and their livestock. The aim of this study was to determine the environmental, animal husbandry and socio-demographic factors associated with occurrence and the pastoral community knowledge of mycobacterial infections at the human-environment-livestock/wildlife interface (HELI) areas in pastoral ecosystems of Uganda.

**Methods:**

Two hundred and fifty three (253) individuals were subjected to a questionnaire survey across the study districts of Nakasongola and Mubende. Data were analyzed using descriptive statistics and multivariable logistic regression analysis.

**Results:**

Humans sharing of the water sources with wild animals from the forest compared to savannah ecosystem (OR = 3.3), the tribe of herding pastoral community (OR = 7.9), number of rooms present in household (3-5 vs. 1-2 rooms) (OR = 3.3) were the socio-demographic factors that influenced the level of knowledge on mycobacterial infections among the pastoral communities. Tribe (OR = 6.4), use of spring vs. stream water for domestic use (OR = 4.5), presence of sediments in household water receptacle (OR = 2.32), non separation of water containers for drinking and domestic use (OR = 2.46), sharing of drinking water sources with wild animals (OR = 2.1), duration of involvement of >5 yrs in cattle keeping (OR = 3.7) and distance of household to animal night shelters (>20 meters) (OR = 3.8) were significant socio-demographic factors associated with the risk of occurrence of mycobacterioses among the pastoral communities in Uganda.

**Conclusion:**

The socio-demographic, environmental and household related factors influence the risk of occurrence as well as pastoralists' knowledge of mycobacterial infections in the pastoral households at the human-environment-livestock/wildlife pastoral interface areas of Uganda.

## Background

Nontuberculous mycobacteria (NTM) are emerging opportunistic pathogens whose role in human and animal disease is increasingly being recognized [1,2]. In the last few decades, the incidence of classical tuberculosis (TB) in man has been on the increase alongside tuberculosis-like disease caused by NTMs [3]. Currently, the major concern is their increasing role as opportunistic pathogens in HIV/AIDS infections and potent immunosuppressive therapies [4]. The apparent increase in the frequency of isolation of NTM's from clinical specimens and the mounting evidence of their involvement in severe pulmonary diseases in man is generating lots of research interest, in as far as the accurate diagnosis and treatment of classical tuberculosis are concerned [3,5].

The health impacts of human-mycobacterial interactions are far complex and likely much broader than currently recognized [1]. The role of the environment (open water sources) as source and livestock/wildlife as reservoirs of infections to man are well documented [1,2,6]. This presents a special health challenge to the pastoral systems in Africa that rely mostly on open natural water sources to meet livestock and human needs. The high interaction at the human-environment-livestock-wildlife interface (HELI) provides an opportunity for the increased contamination of the natural water sources and the transmission of NTMS to livestock and humans. Reports from developing countries show NTM incidence rates varying between 2-20% [5].

Pastoral communities in Uganda occupy the semi arid cattle corridor that extends diagonally from the pastoral Ankole in the south west through central Uganda to the Karamoja region in the North East [7]. These semi-arid pastoral areas in general suffer from poor quality pastures and seasonal water availability [8]. In these areas, valley dams and valley tanks are the major sources of water for humans, domestic and wild animals. The sharing of these stagnant and other natural water sources by pastoral communities with livestock and wildlife presents another dimension in the epidemiology of NTMs in this high risk group. Water is considered the primary source of *Mycobacterium avium *complex (MAC) infections in humans, while domestic and wild animals may be reservoirs [9].

A 'one health approach' study conducted in the pastoral areas of Karamoja in Uganda involving isolation and characterization of mycobacteria from lymph nodes from humans (cervical lymphadenitis patients) and their livestock, found NTMs as important causes of morbidity[10,11]. Isolation and characterization of mycobacteria causing cervical lymphadenitis in pastoral communities in Karamoja region showed that 51% of the cases were caused by NTMs, of which the *Mycobacterium avium-intracellulare *Complex (MAC) accounted for 55 percent [11]. While a parallel study of 61 tuberculous lesions from slaughter cattle in the same region yielded 37 isolates of which NTM accounted for 48.6% (18/37) [10]. It is well known that direct animal to human transmission of NTM is of less significance in the epidemiology of NTMs. Therefore, the occurrence of infections by same agents in both pastoral cattle and communities was strongly suggestive of the primary role of environment. To address the problem of NTM infections in HELI areas in the pastoral systems, it requires broader approaches of 'Ecosystem health' that considers studying all the three components simultaneously. The 'Ecosystem health' approach is justified because; the incremental knowledge generated is higher compared to three separated human, animal and environment health studies.

The aim of this study was to determine the environmental, animal husbandry and socio-demographic factors associated with occurrence and the pastoral community knowledge of mycobacterial infections at the human-environment-livestock/wildlife interface areas in pastoral areas of Uganda.

## Methods

### Study area

**Figure 1:**
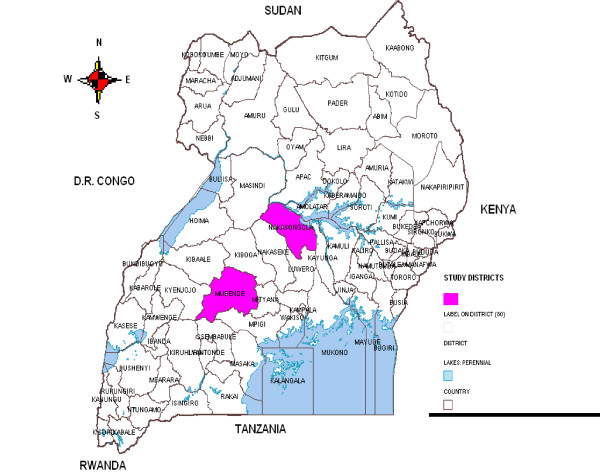
**Map of Uganda Showing the locations of the Study districts**.

The study was conducted in Nakasongola and Mubende districts of Uganda. Both districts are located in the cattle corridor in central Uganda (Figure 1). Mubende district (altitude-1323 m) is located at latitude 0°35' 21N s and longitude 31°21' 36E while Nakasongola district (altitude -1,079 m) is located at latitude 1°18' 32N and longitude 32°27' 23E. Nakasongola is one of the cattle corridor districts that suffers from persistent droughts [12]. Likewise, Nakasongola district was selected based on a high number of pastoral cattle population and the results from a previous study [10], in which mycobacteria outside the *M. tuberculosis *complex (MOTT) was found high in lymph nodes of slaughter cattle originating from there. Mubende, a district with high level of disease, poverty and limited clean water sources was chosen for comparative purposes [13]. Kiyuni and Madudu sub-counties in Mubende district and Nabiswera and Lwampanga sub-counties in Nakasongola were selected mainly based on similarities and relevance of their ecosystems to the purpose of the study such as existence of shared water sources, high degree of interaction between human-domestic animal and wildlife, as well as status of social service delivery system. In these areas, livestock, wildlife and communities often share water from valley dams, valley tanks, seasonal ponds and streams.

### Sample size determination

Sample size determination was based on previous studies on prevalence, mortality associated with tuberculosis in HIV infected patients in rural Uganda [14]. It was also based on the high level of non tuberculous mycobacteria (NTM) causing caseous lymphadenitis in pastoral communities in Karamoja region of Uganda [11]. The two studies showed the tuberculosis prevalence of 2-7% (mean = 4.5%). Based on the district state of environment National Environment Management Authority (NEMA), the population in the above four sub-counties was estimated to be 43,000[12,13]. Based upon the simple assumption that an absolute minimum of 10 individuals with TB in the study population, we opted for 250 as a necessary minimum sample size, enabling also a reliable comparison between population factors at a prevalence of 10-20% Ausvet Epitools [15]. With the existing logistics, facilities and specified time period, a total of 253 study individuals from households in the study areas were interviewed.

### Study design and sampling strategy

The primary sampling units was sub-county, while the ultimate sampling unit was village. Selection of household from list of households generated in collaboration with the community local leaders' in the respective sub-counties was based on systematic approach. A total of 253 individuals, each a proxy for a household, were selected based on their social role in the household and participation in the local community affairs. Those individuals/households not considered in this study were reserved for Focus Group Discussions (FGD) and community discussion group participation in the study.

### Case definition

#### Assessment of occurrence

Definition of TB patient cases as an outcome variable for TB and other mycobacterial infections status was based on retrospective health record of the interviewee or individuals in his/her household in the last 10 years. Once a previous TB patient was identified, permission was sought from both the said individual and the health centre that managed their case, for access to the records of confirmed diagnosis. Confirmed diagnosis of individual with tuberculosis was based on positive sputum smears on Ziehl-Nielsen stain (ZN+) and chest X-ray, while for mycobacterial caseous lymphadenitis, on biopsies positive on ZN stain. No culturing was done in these health facilities.

#### Assessment of degree of knowledge on mycobacterial infections among the pastoral community

During the interviewing exercise, the following aspects were assessed: 1) Definition of tuberculosis or any mycobacterial infections 2) Causes 3) Symptoms 4) Modes of transmission and 5) Prevention or control measures employed in tuberculosis or any mycobacterial infections. The ability of the respondent to clearly mention in local language or English, any of the aspects above would qualify the respondent as more knowledgeable: On the other hand, failure to mention any of the aspects above would categorize the respondent as less knowledgeable.

### Possible source of bias and the way handled

A trained interviewer administered a standardized questionnaire to each study participant in the local languages (Runyankore, Ruruli, Runyoro or Luganda). Using the designed and pretested questionnaire as a tool for data collection, standardization across all the study districts was done, by translating and re-translating the questionnaire from English to local languages and back to English. This allowed standardization of responses across all different ethnic groups involved in the study. Secondly, recruitment of two well-oriented medical research assistants in order to ensure over all technical supervision, correct filling of the questionnaire forms and appropriate data compilation and entry was done. A trained interviewer administered a questionnaire during the interviews to capture outcomes of interest -i.e. the knowledge and occurrence of mycobacterial infections.

Socio-demographic information relevant to the study purpose, as well as, contacts with animals, water sources and associated factors such as water availability, locations, and distances from household to water sources and sharing of water sources with domestic and wild animals were captured from the respondents.

At household level, information on the quality of dwelling housing in terms of construction details, availability of rooms and ventilation (windows), and the average occupancy per room was captured. Methods employed by the community in management of clinical or overt mycobacterial infections were also documented. Location of and access to health services including both public and private health services were recorded. Environmental soils were collected from cattle night enclosures (kraals) and places where pigs are tethered or housed near homesteads for mycobacterial analysis. In addition, a water source used by the household for collection of drinking and domestic use was collected. The type, frequency of cleaning and replenishment of the household water receptacles was recorded. Water from the bottom of the household containers was also sampled. Collected samples were appropriately packaged, labelled, stored at -79°C. The samples were submitted to the laboratory at the National Veterinary Institute, Oslo, Norway for culturing, identification and characterization of the isolates. Results from the biological samples will be presented in a subsequent paper.

### Data analysis

Statistical analysis was carried out using Stata version 10/SE for windows (StataCorp, College Station, TX). Summary statistics of the explanatory variables with respect to community's knowledge and occurrence of mycobacterial infections were carried out using the tab command of Stata. The outcomes of interest were: a) the medical history of tuberculosis and other related mycobacterial infections by isolation of mycobacteria in the cervical lymph nodes and presence of a unilateral single or multiple painless lumps, mostly located in posterior cervical or supra-clavicular region in any member of household or community, b) community knowledge about the mycobacterial infection.

The proportion of the respondents with outcome of interest was estimated using survey commands and descriptive statistics in Stata. Table 1 shows exposure variables that were considered for the initial screening, those with p ≤ 0.25 in univariable analyses which were considered for inclusion in the final model. The final multivariable logistic regression model was built with backward elimination procedures [16]. The validity of the models in explaining usefulness of the explanatory variables and in predicting the outcome variables was assessed using Hosmer-Lemeshow goodness-of-fit test, Receiver Operating Characteristic curve(ROC) and test property statistics (sensitivity and specificity).

**Table 1:** Environmental, animal husbandry, household and socio-demographic factors associated with occurrence of and respondent's knowledge on mycobacterial infections among the pastoral communities in the districts of Nakasongola and Mubende, Uganda.

Exposure variable	Levels
District	Nakasongola and Mubende
Subcounty	Kiyuni, Madudu, Nabiswera and Lwampanga
Sex	Male, Female
Tribe	Baganda, Banyoro, Bakiga, Baruli, Banyankole and Basoga
Marital status	Married, Single, Divorced
Family sizes	<10, 10-20 and 20-30 persons
Occupation	Pastoralist, Peasant, Hunter, Business man, Herdsman
Role in cattle or any animal management	None, Herding, Milking, Watering
Source of drinking water	Stream, Borehole, Valley dam, Pond, Spring
Source of water for domestic use	Stream, Borehole, Valley dam, Pond, Spring
Receptacle for storing water for daily use	Clay, Gourd, Plastic, Pond, Spring
Sharing water sources with other animals	Yes/No
Occurrence of wildlife at the water source.	Yes/No
The type of Wildlife-water source.	No recall, Monkey, Rabbits
Use of separate receptacle in drinking and domestic water storage.	Yes/No
Presence of the sediment in the domestic water receptacle	Absent, Always, Sometimes.
Frequency of cleaning the container	Daily, twice a week, once week
Water related human diseases	Diarrhoea, Cough, Worms, Malaria
Average number of rooms present in household	1-2, 3-5 and > 6 rooms
Average number of windows	0, 1-2, 3-5 and >6 windows
Number of people sleeping together in a single room	1-3, 3-5 and 6-9 people
The period drinking water lasts in the container	A few hours, 1-3 days
Keeping animals in the human shelter at night	Yes/No
Diagnosis of TB adenitis and other mycobacterioses	Not diagnosed, Diagnosed
Diagnosis and treatment done following TB illness	Yes/No
Heard/seen signs of adenitis	Yes/No
Site of adenitis noticed in the patient within the family or community	No recall, Cervical, Inguinal

### Ethical approval

Scientific and ethical clearance to conduct this study was obtained from the Uganda National Council for Science and Technology (UNCST). The study was reviewed by the research ethics committee and found to be scientifically and ethically satisfactorily and it was approved with a reference: H337.

## Results

The multivariable logistic regression analysis showing the influence of socio-demographic, environmental and household related factors on community's knowledge on the mycobacterial infections among pastoralists is presented in table 2. Assessment of model fit to observed data showed insignificant difference between the observed and predicted values (HL (χ^2^) = 4.98; P = 0.76). Likewise, the ability of the model to reasonably predict community knowledge about mycobacterial diseases is highly reliable (ROC = 0.93).

**Table 2:** Multivariable logistic regression analysis of socio-demographic factors that associated with the level of knowledge on mycobacterial infections among the pastoral communities in the districts of Nakasongola and Mubende, Uganda.

Risk factor	Level	Odds Ratio 95% [CI]	P-value
Tribe	Banyankole vs. Baganda	7.9 [1.5-42.4]	0.016
The type of wildlife^a ^seen at the water source.	Forest ecosystem vs. Savannah.	0.3 [0.1-0.7]	0.008
The number of rooms present in household	3-5 vs. 1-2 rooms per house.	3.3 [1.2-9.1]	0.021

^**a **^comparison of wildlife from savannah and forest ecosystems.

The Banyankole pastoral communities showed a high level of knowledge about mycobacterial infections relative to the Baganda tribe (OR = 7.9). The majority of the respondents perceived that presence of forest wild animals and night overcrowding of people sleeping in windowless rooms as risk factors (OR = 3.3) for acquiring infections among the pastoral communities. Pastoralists were found to associate mycobacterial infections with water contamination from animals of forest ecosystem than savannah group of wildlife animals (Table 2). Pastoralists who didn't share water sources for drinking and domestic purposes with animals were equally less knowledgeable of mycobacterial infections. According to the respondents, the forest ecosystem includes wild birds, wild pigs, monkeys and other primates. The savannah ecosystem animals include the ruminant group such as the antelope family.

The influence of socio-demographic, environmental and household related factors on the occurrence of tuberculosis and other mycobacterial infections among pastoralists is presented in table 3. Basoga pastoralists were at higher risks for acquiring mycobacterial infection (OR = 6.4) compared to Baganda. Pastoralists using open sources of water (stationary water) were much more likely to report mycobacterial infections than those getting water from streams (flowing water). Presence of sediments in domestic household water containers was not linked directly to mycobacterial infections. Shared water collection points (Water used for other purposes e.g. drinking, for animals etc), was highly linked to an increase in the risk of acquiring mycobacterial infections. Rearing of cattle and other animals over a long period of time and absence of wild animals at water sources reduced exposure of humans to mycobacterial infections.

**Table 3:** Multivariable logistic regression analysis showing the association between socio-demographic factors and occurrence of mycobacterioses among the pastoral communities in the districts of Nakasongola and Mubende, Uganda.

Risk factor	Level	Odds Ratio [95% CI]	P-value
Tribe	Basoga vs. Baganda	6.4[1.56-26.4 ]	0.01
The source of water for use in households	Spring^y ^vs. Stream^z^	4.5[ 1.7-11.9]	0.002
Presence of sediment in the water container.	Yes vs. No.	0.43[0.24-0.79]	0.006
Separation of water containers for drinking and domestic uses.	Yes vs. No.	2.46[1.3-4.4 ]	0.004
Sharing of water sources with wild animals.	Yes vs. No.	0.47[0.25-0.9 ]	0.024
Duration of involvement in rearing cattle and other cattle related activities.	> 5 yrs vs. <5 yrs	0.27[0.089-0.8 ]	0.021
Distance of Kraal/animal shelter from the household	>20 meters vs. < 20 m	3.8[1.14-12.5 ]	0.029

^y ^means stagnant water sources. The valley dams and tanks are constituted in similar process.

^z ^means flowing or stream water. Streams water flows continuously.

Results also showed that pastoral household members residing in close proximity (< 20 meters) to cattle night enclosures or pig tethering area were at higher risk (OR = 3.8) of acquiring mycobacterial infection than those living at greater distances. Evaluation of model fit to the data, showed its high validity (HL (χ^2^) = 7.85; P = 0.25). The reliability of the model to predict the presence of mycobacterioses was relatively high (ROC = 0.74)

## Discussion

To our knowledge, this is the first study focusing on the level of knowledge and occurrence of mycobacterial infections in the human-environment - livestock/wildlife interface among the central Ugandan pastoral communities. The study explored the relationship between humans sharing the limited water sources with animals (both domestic and wild). Findings from this study reveal tribe as one of the socio-demographic determinants that play a significant role in both outcomes of this study. The Banyankole had a high degree of knowledge about mycobacterial infections compared to any other tribe in the study areas. The reason for this is not fully understood, however the Banyankole pastoral communities have kept animals for a longer time than Baganda communities [17]. It is noted that the Banyankole tribe has predominantly occupied the cattle corridor districts which are arid and semi-arid areas of Uganda characterised by extremely difficult weather and scarce water sources[18,19]. It is likely that their bitter experience with zoonoses acquired from their cattle over the years have given them the opportunity to learn the causes, spread mechanisms, prevention and control strategies of mycobacterial infections. Most of the Basoga communities occupy the agro-pastoral setting with few heads of cattle and other livestock. Lack of experience and being new in the pastoral farming system is likely to be one of the contributory factors to what was observed in the study.

This study showed that the pastoral communities were knowledgeable of effects of overcrowding of people in sleeping rooms. While the mechanisms of mycobacterial disease spread are known [20], social distances and degree of organisation are known to influence contacts thus increasing the distribution and transmission tuberculosis and other mycobacterioses within a population [21]. Overcrowding in poorly ventilated rooms, poor sanitation and absence of or distant located health care which are common findings in pastoral rural populations and are known drivers in the spread of mycobacterial infections in developing countries [22,23].

It is important to notice that wild animal tuberculosis represents a permanent reservoir of infections and poses a serious threat to control and elimination programs [24], while contaminated water sources provide the foci of mycobacterial infections in the humans and animals [9,25]. This greatly increases the risk of mycobacterial infections in the susceptible species across humans, wild and domestic animal populations. The natural water sources support the formation of *M. avium *and *M. intracellulare *in biofilms [26,27]. This leads to the persistence of mycobacteria in the aquatic environment

The study revealed that humans extensively share open water and the only available water resources mainly during drought with domestic and wild animals (wild boar, birds, monkeys, baboons, and antelopes). Therefore, there is an increased risk of NTM transmission between wild animals and the pastoral communities. These pastoralists concerns about mycobacterial transmission tied up with findings of *M. tuberculosis *transmission between humans and elephants [28]. Similar roles of wildlife in the epidemiology mycobacterial infection have also been reported in wild boar, seals, rhinoceros, and elk among the wild animals [29-33].

The presence of sediments in domestic water receptacles was not associated with occurrence of mycobacterial infections in households. Contrary to this, sediments are known to provide foundation for bio-film formation [27]. However, sediments in this study were closely linked to fast moving or turbulent stream water; a source we earlier stated had less epidemiological role in household infections with mycobacteria. Sharing of receptacles for water storage (drinking and other uses) was closely associated with occurrence of mycobacterioses. Although direct relationship between this practice and infection can not be explained by this study alone, we believe that this practice is a proxy for socioeconomic status of the households involved, that the poor families could not afford receptacles to keep drinking water safe and separate from water used for other activities. Besides, these households were also identified as obtaining water from risky sources.

Furthermore, the study revealed that compared to the pastoralists who herded animals for a shorter period of time, the time taken in herding was key in the occurrence of mycobacterial infections in pastoral communities. There is a high likelihood of acquiring immunity in individuals with longer exposure to mycobacterioses. Individuals with prior exposure to mycobacterial antigens present these antigens to the T-lymphocytes which helps in the development of a strong immune response among people who have herded animals over a longer period of time [34].

Under the pastoral setting, cattle are an integral part of human social life [24]. Cattle enclosures are also located very close to human dwellings to deter livestock theft. Results indicate that pastoralist constructing animal enclosures close to their homesteads had an increased risk or history of mycobacterial infections. In addition, across all the pastoral tribes, there was an inverse relationship between distance of the location of the kraal or any other animal shelter from the homesteads and the history or risk of acquiring mycobacterial infections in households. This inverse relationship is likely to be associated with the aerosol spread of the pathogens causing tuberculosis from the domestic animals to humans [24,34].

According to the evidence presented in [35], cattle to human aerosol transmission of *Mycobacterium bovis *rarely occur. Besides, high occupational risk exposed individuals have been confirmed in other developing African countries e.g. Burkina Faso [36].

## Conclusions

This study has shown that the level of knowledge and degree of occurrence of mycobacterial infections among the pastoral communities is greatly influenced by socio-demographical factors, environmental, intra and extra household factors. The study has further indicated that there is a close interaction between and within species that aid the transmission of mycobacterial infections across the humans, animals and the environment.

Overall, the socio-demographic, environmental and household related factors influence pastoralist's knowledge as well as risk of occurrence of mycobacterial infections in the pastoral households at the human-environment-livestock/wildlife pastoral interface areas of Uganda.

## Competing interests

The authors declare that they have no competing interests.

## Authors' contributions

CK contributed to the design, data collection, drafting and writing of the manuscript. AM contributed to data collection, data analysis and drafting of the manuscript. SO contributed to data analysis and drafting of the manuscript, MM contributed to design, data analysis and drafting of the manuscript, DB contributed to the data analysis and drafting of the manuscript. JOA contributed to the conception, design and supervision, and drafting and writing of the manuscript. ES contributed to the acquisition of funds, drafting of the manuscript and JO: contributed to the conception, design and supervision, drafting and writing of the manuscript. All authors have read and approved the final manuscript.

## Pre-publication history

The pre-publication history for this paper can be accessed here:

http://www.biomedcentral.com/1471-2458/10/471/prepub

## References

[B1] Primm TP Lucero CA Falkinham JO Health impacts of environmental mycobacteria Clin Microbiol Rev 2004 17 1 98 106 10.1128/CMR.17.1.98-106.2004 14726457 PMC321467

[B2] van Ingen J Boeree MJ Dekhuijzen PN van Soolingen D Environmental sources of rapid growing nontuberculous mycobacteria causing disease in humans Clin Microbiol Infect 2009 15 10 888 893 10.1111/j.1469-0691.2009.03013.x 19845700

[B3] Mawak J Gomwalk N Bello C Kandakai-Olukemi Y Human pulmonary infections with bovine and environment (atypical) mycobacteria in jos, Nigeria Ghana Med J 2006 40 4 132 136 17496986 10.4314/gmj.v40i3.55268 PMC1868006

[B4] Corti M Palmero D Eiguchi K Respiratory infections in immunocompromised patients Curr Opin Pulm Med 2009 15 3 209 217 10.1097/MCP.0b013e328329bd2c 19276812

[B5] Idigbe EO Anyiwo CE Onwujekwe DI Human pulmonary infections with bovine and atypical mycobacteria in Lagos, Nigeria J Trop Med Hyg 1986 89 3 143 148 3534283

[B6] Chilima BZ Clark IM Floyd S Fine PE Hirsch PR Distribution of environmental mycobacteria in Karonga District, northern Malawi Appl Environ Microbiol 2006 72 4 2343 2350 10.1128/AEM.72.4.2343-2350.2006 16597928 PMC1449038

[B7] United Nations Development Programme in Uganda http://www.undp.or.ug/visited on 4/2/10

[B8] Uganda: Water scheme proposed for parched for Karamoja-News-professional resources-PreventionWeb.net http://www.preventionweb.net/english/professional/news/v.php?id=7973 Visited 5/02/10

[B9] Biet F Boschiroli ML Thorel MF Guilloteau LA Zoonotic aspects of Mycobacterium bovis and Mycobacterium avium-intracellulare complex (MAC) Vet Res 2005 36 3 411 436 10.1051/vetres:2005001 15845232

[B10] Oloya J Kazwala R Lund A Opuda-Asibo J Demelash B Skjerve E Johansen TB Djønne B Characterisation of mycobacteria isolated from slaughter cattle in pastoral regions of Uganda BMC Microbiology 2007 7 95 10.1186/1471-2180-7-95 17961243 PMC2140064

[B11] Oloya J Opuda-Asibo J Kazwala R Demelash AB Skjerve E Lund A Johansen TB Djonne B Mycobacteria causing human cervical lymphadenitis in pastoral communities in the Karamoja region of Uganda Epidemiol Infect 2008 136 5 636 643 10.1017/S0950268807009004 17599779 PMC2870852

[B12] Anonymous District State of Environment Report for Nakasongola District 2004 67

[B13] Anonymous District State of Environment, Mubende District 2004 129

[B14] Moore David Liechty Cheryl Ekwaru Paul Were Willy Mwima Gerald Solberg P Rutherford George Mermin J UGANDA: Prevalence, Incidence and Mortality Associated with Tuberculosis in HIV-Infected Patients Initiating Antiretroviral Therapy in Rural Uganda AIDS 2007 21 6 713 719 10.1097/QAD.0b013e328013f632 17413692

[B15] Epitools http://epitools.ausvet.com.au/content.php?page=SampleSize Visited 25/1/10

[B16] Dohoo I Martin W Stryhn H Veterinary Epidemiologic Research. AVC inc, Charlottentown, Canada 2003 35 42

[B17] Banyankole http://www.everyculture.com/wc/Tajikistan-to-Zimbabwe/Banyankole.html visited 5/02/10

[B18] Mpairwe D Mutetikka D Kiwuwa GH Mugerwa S Owoyesigire B Zziwa E Peden D Options to improve livestock-water productivity (LWP) in the cattle corridor within the White Nile sub-basin in Uganda 2008 1 5

[B19] Wurzinger M Ndumu D MO A Solkner J Lifestyle and herding practices of Bahima pastoralists in Uganda Afr J Agric Res 2008 3 542 548

[B20] Fratazzi Candida Manjunath N Robert D Arbeit RD Carini Claudio T A Gerken Ardman Blair Eileen Remold-O'Donnell Remold HG A Macrophage Invasion Mechanism for Mycobacteria Implicating the Extracellular Domain of CD43 2000 192 2 183 191 10899905 10.1084/jem.192.2.183 PMC2193255

[B21] Jaramillo E Tuberculosis and Stigma: Predictors of Prejudice against People with Tuberculosis J Health Psychol 1999 4 71 79 10.1177/135910539900400101 22021435

[B22] Triggle DJ Medicine in the 21st century or pills, politics, potions, and profits: where is the public policy? Drug Dev Res 2003 59 269 291 10.1002/ddr.10282

[B23] Kriege J L HD Housing and health: time again for public health action Am J Public Health 2002 92 757 768 10.2105/ajph.92.5.758 PMC1447157 11988443

[B24] Cosivi O Grange JM Daborn CJ Raviglione MC Fujikura T Cousins D Robinson RA Huchzermeyer HF de Kantor I Meslin FX Zoonotic tuberculosis due to Mycobacterium bovis in developing countries Emerg Infect Dis 1998 4 1 59 70 10.3201/eid0401.980108 9452399 PMC2627667

[B25] Collins CH Grange JM YM D Mycobacteria in water Applied Bacteriology 1984 57 193 211 10.1111/j.1365-2672.1984.tb01384.x 6389461

[B26] Carter G Wu M Drummond DC Bermudez LE Characterization of biofilm formation by clinical isolates of Mycobacterium avium J Med Microbiol 2003 52 Pt 9 747 752 10.1099/jmm.0.05224-0 12909649

[B27] Falkinham JO Norton CD LeChevallier MW Factors influencing numbers of Mycobacterium avium, Mycobacterium intracellulare, and other Mycobacteria in drinking water distribution systems Appl Environ Microbiol 2001 67 3 1225 1231 10.1128/AEM.67.3.1225-1231.2001 11229914 PMC92717

[B28] Michalak K Austin C Diesel S Bacon JM Zimmerman P Maslow§ JN Mycobacterium tuberculosis infection as a Zoonotic Disease: Transmission between Humans and Elephants Emerg Infect Dis 1998 4 282 287 10.3201/eid0402.980217 PMC2640151 9621200

[B29] Thoen CO Tuberculosis (Revised 1995). Zoonosis updates J Am Vet Med Assoc 1995 2 155 158

[B30] Thompson PJ Cousins DV Gow BL Collins DM Willamson BH Dagnia HT Seals, seal trainers, and mycobacterial infection Am Rev Respir Dis 1993 147 164 167 8420412 10.1164/ajrccm/147.1.164

[B31] Dalovisio JR Stetter M Mikota-Wells S Rhinoceros' rhinorrhea: cause of an outbreak of infection due to airborne Mycobacterium bovis in zookeepers Clin Infect Dis 1992 15 598 600 1420672 10.1093/clind/15.4.598

[B32] Fanning A Edwards S Mycobacterium bovis infection in human beings in contact with elk (Cervus elaphus) in Alberta, Canada Lancet 1991 338 1253 1255 10.1016/0140-6736(91)92113-G 1682654

[B33] Zanella G Durand B Hars J Moutou F Garin-Bastuji B Duvauchelle A Fermé M Karoui C Boschiroli ML Mycobacterium bovis in wildlife in France J Wildl Dis 2008 44 99 108 18263825 10.7589/0090-3558-44.1.99

[B34] Ayele WY Neill SD Zinsstag J Weiss MG Pavlik I Bovine tuberculosis: an old disease but a new threat to Africa Int J Tuberc Lung Dis 2004 8 8 924 937 15305473

[B35] Torgerson PR Torgerson DJ Public health and Bovine tuberculosis: what's all the fuss about? Trends in Microbiology 2010 18 2 67 72 10.1016/j.tim.2009.11.002 19944609

[B36] Vekemans M Potential source of human exposure to *Mycobacterium bovis *in Burkina Faso, in the context of HIV epidemic Clinic microbial infect 1999 5 617 621 10.1111/j.1469-0691.1999.tb00418.x 11851692

